# Association of iodized salt with goiter prevalence in Chinese populations: a continuity analysis over time

**DOI:** 10.1186/s40779-017-0118-5

**Published:** 2017-03-21

**Authors:** Zhen Liang, Chen Xu, Yong-Jun Luo

**Affiliations:** 1 Department of Military Medical Geography, College of High Altitude Military Medicine, Third Military Medical University, Chongqing, 400038 China; 2 Battalion 5 of Cadet Brigade, Third Military Medical University, Chongqing, 400038 China; 3 Key Laboratory of High Altitude Medicine (PLA), Third Military Medical University, Chongqing, 400038 China

**Keywords:** Iodine deficiency disorders (IDD), Iodized salt, China

## Abstract

**Background:**

Iodine deficiency disorders (IDD) refer to diseases that are caused by insufficient iodine intake, and the best strategy to prevent IDD is the addition of iodine to dietary salt. Because iodine deficiency is a common cause of goiter, the prevalence as effectively controlled after the implementation of universal salt iodization (USI) in China. However, there is substantial controversy as to whether the incidence of thyroid disorders is related to iodized salt intake. Therefore, we aimed to clarify whether the risk of goiter can be promoted by USI.

**Methods:**

A longitudinal continuous study based on the national monitoring results of IDD in China was performed for 3 consecutive years. We recorded the following indicators of IDD from 31 provinces: goiter number, two degrees of goiter (the degree of goiter severity) and cretinism (three endemic diseases), iodized salt intake, median urinary iodine concentration (UIC), soil iodine content and coverage rates of iodized salt. One-way Analysis of Variance (ANOVA) and linear regression analyses examined the differences between the three groups and correlations, respectively. Data were collected from the Chinese national IDD surveillance data in 2011-2013, and the background values of Chinese soil elements were published in 1990.

**Results:**

A reference male’s daily intake of maximum iodine was 378.9 μg, 379.2 μg and 366.9 μg in 2011, 2012, and 2013, respectively. No statistical association between daily iodized salt intake and the three endemic diseases was observed in 2011-2013 (*P* > 0.05). No association was observed between daily iodized salt intake and the UIC of children in 2011 (*P* > 0.05). Linear regression revealed no significant correlation between the soil iodine content and three endemic diseases. The present study indicated no difference in the daily iodized salt intake in each province during three years (*F* = 0.886, *P* = 0.647). The coverage rate of iodized salt remained above 98.7%, and goiter rates were stable in 2011-2013.

**Conclusion:**

There was no significant association between iodized salt intake and the three endemic diseases, suggesting that the current nutrition level of iodized salt did not cause the high goiter prevalence.

## Background

Iodine is an essential trace element for the synthesis of thyroid hormones containing thyroxine and tri-iodothyronine [1, 2]. Iodine in the soil can also be absorbed into the human food chain through plants, which may affect the occurrence of endemic goiter [3–5]. Iodine deficiency and excess intake lead to iodine deficiency disorders (IDDs) and iodine excess disorders (IEDs), respectively [1, 2]. Several risk factors, such as tobacco smoking, genetic factors, gender, alcohol and age, are associated with an increased risk of IDD, but iodine deficiency is realized as an important risk factor [6, 7]. Approximately 30 countries worldwide suffer from iodine deficiency, and 10 countries exhibit excessive iodine intake [8]. Iodine deficiency has widespread implications, including endemic goiter, cretinism, miscarriage and abnormal brain development in children [9, 10]. Moreover, IED can lead to hyperthyroidism, autoimmune thyroid disease and thyroid cancer [11]. The best strategy to prevent IDD is salt iodization, which is the most effective, economical and convenient measure for iodine fortification that is used in most countries [12].

These serious situations could affect a child’s growth and lead to mental retardation and dysgnosia as well as reduce the survival rates of children. The primary clinical manifestation was cretinism [13]. The USI program of China was launched in 1995 to prevent IDD, and it had a significant effect [14]. The level of iodine nutrition of the entire nation significantly improved, and IDD as effectively controlled. An assessment from the Chinese Ministry of Health in 2000 announced that China had completely eliminated IDD.

However, the health hazard of iodine excess gradually received attention after the implementation of USI in 1995. The UIC of school-aged children simultaneously sharply increased and reached 330 μg/L in 1997 and 306 μg/L in 1999 [15, 16], which was beyond the recommended levels of 100.0 to 199.9 μg/L and more than adequate (200.0 to 299.9 μg/L) according to the World Health Organization (WHO) [17]. Endocrinologists reported that the prevalence of goiter exhibited an increasing trend that was closely related to the amount of commonly consumed iodized salt [18–20]. Therefore, whether it was scientifically sound and essential to sustain the USI program throughout China was heavily debated [21–24]. There have been many cross-sectional studies, but these analyses suffered a lack of continuity over time.

## Methods

### Study setting

We collected national IDD surveillance data from three recent years and the content of iodine in soil from surveillance data in 1990. One-way Analysis of Variance (ANOVA) and linear regression analyses were used to examine differences between the three groups and correlations, respectively. Coverage rates of iodized salt from 1995, 1997, 1999, 2002, 2005 and 2011 to 2013 were selected to assess national iodized salt consumption levels. Ethical approval was obtained from the ethical committee of the Third Military Medical University in China. Written informed consent was obtained from every participant prior to enrollment.

### Diagnostic criteria for thyroid disease

Thyroid ultrasonography was performed using a 7.5 MHz transducer to diagnose goiter. Experienced radiologists from the local Centers for Disease Control and Prevention measured the thyroid volumes in the 31 provinces. The thyroid lobe volume was calculated by measuring the depth (d), width (w) and length (l) of each lobe by the following formula: V (ml)  =  0.479 × d × w × l (mm)/1000. The thyroid volume was recorded as the sum of both lobes. The normal volumes for adult males and females are less than 25.6 ml and 19.4 ml [25], respectively.

### Data collection and sampling

We collected consecutive national statistics data from August 2012 to September 2014. A probability proportional-to-size (PPS) cluster sampling was applied in the national IDD surveillance data.

### Cases of IDD collection and processing

We collected data on the prevention and treatment of IDD in Chinese provinces for three consecutive three years (2011, 2012 and 2013). Provincial level indexes of the 31 provinces were applied when individual provinces lacked data in the statistical yearbook. The total population of each province is the sum of the number of diseased counties. The number of goiter cases in three years (not including Shanghai, Guangdong and Qinghai) as well as two degrees of goiter cases (not including Tianjin, Shanghai, Jiangsu, Guangxi, Qinghai and Guangdong in 2011 and 2012; Tianjin, Shanghai, Guangdong, Guangxi, Hainan and Qinghai were not included in 2013) and cretinism cases in three years (not including Beijing, Tianjin, Shanghai, Jiangsu, Hainan, Tibet and Guangdong) were collected. The prevalence of three endemic diseases rates (shown in ‱) were calculated using the following formula: the number of prevalent cases divided by the total population of each province in the diseased counties.

### The index related to IDD

We chose the actual number of annual sales of iodized salt and non-iodized salt in 2011, 2012 and 2013. The type of salt was defined as follows: non-iodized salt, salt iodine less than 5 mg/kg, qualified iodized salt, salt iodine 20-30 mg/kg, others, and unqualified iodized salt [26]. The ratio of the number of iodine salts to the total number of salts represented the coverage rate of iodized salt in 2011-2013 (1995, 1997, 1999, 2002 and 2005 were five times the coverage rate of iodized salt collected from summary analysis of monitoring data of IDD in the entire country) [15, 16, 27–29]. The UIC (μg/L) in school-aged children who were 8 to 10 years of age was determined in 2011. We did not assess surveillance data of the UIC in 2012 and 2013 because they were not available. We collected surveillance data of the soil iodine content in 1990 because background values of soil iodine did not significantly change in China [30]. All data came from the China Statistical Yearbook of Health and Family Planning issued in 2012, 2013 and 2014, which was conducted by the Peking Union Medical College Publishing House. The China Background Value of Soil Element was published by the China Environmental Science Press in 1990.

### Statistical analyses

We used Microsoft Office Excel 2007 software to establish the database and create a line chart to reflect the coverage rates of iodized salt in each province. Data processing and statistical analyses were performed using SPSS statistics version 18.0. One-way ANOVA compared the three years of actual sales of iodized salt to identify significant differences. Pairwise comparisons were not performed if the null hypothesis was accepted (*P* > 0.05). Correlations between the incidence of the three endemic diseases, daily iodized salt intake, the UIC (μg/L) of children and the content of iodine in soil were analyzed using regression analyses to confirm the quantitative relationship between two or more variables. The results analyzed the value of Sig. (significance), which was the significance coefficient of the regression relationship and the actual significance probability of the *F* value, named the value of *P*. All tests of significance were two-tailed, and *P* < 0.05 was considered significant.

## Results

### Data summary

Table 1 presents the general features of three consecutive years of data, including 2011, 2012 and 2013. The total population with disease in the county was reduced by 2304.1 ten thousand people from 2011 to 2013 and the cases of three endemic diseases continue to decline. Per capita daily intake of iodine ranged from 244.6 to 379.2 μg/d, which exceeds the recommended dietary allowance of iodine 150 μg/d according to the WHO/UNICEF/ICCIDD [31].

**Table 1:** The general features of three consecutive years of data

Item	2011	2012	2013
The total population with disease in the county (ten thousand people)	132,636.8	130,498.5	130,332.7
The number of goiter cases (‱^a^)	4,897,422 (36.923,6)	4,800,287 (36.784,2)	4,624,814 (35.484,7)
The number of two degree of goiter cases (‱^a^)	230,621 (1.738,79)	224,834 (1.722,9)	219,635 (1.685,2)
The number of cretinism cases (‱^a^)	107,219 (0.808,4)	101,820 (0.780,2)	92,219 (0.707,6)
UIC (μg/L) in school-aged children 8 to 10 years of age	226.5	—	—
Annual sales of iodized salt (tons)	6,115,769	6,024,514	5,820,098
The number of iodine salt copies	812,617	834,523	797,154
The number of non-iodized salt copies	10,283	9978	8546
Coverage rate of iodized salt (percent)	98.750,4	98.818,5	98.939,3
Per capita daily intake of iodized salt (g)	12.63	12.64	12.23
Per capita daily intake of iodine (μg)	252.6-378.9	252.8-379.2	244.6-366.9

‱^a^: Indicates 1/10000 (‱)

### Correlation between daily iodized salt intake, three endemic diseases and the UIC of children

Table 2 shows the associations of iodized salt intake with the rate of goiter, two degrees of goiter and cretinism in 2011, 2012 and 2013. Moreover, we evaluated the correlation between iodized salt intake and UIC in school-aged children 8 to 10 years of age in 2011. The daily iodized salt intake was not associated with the three endemic diseases or UIC because there was no significant relationship between these data (*P* > 0.05).

**Table 2:** Correlations between the daily iodized salt intake, three endemic diseases and UIC of children

Dependent variable	B	Std. Error	Beta	*t* value	*P* value
2011					
The rate of goiter	-2.738	4.472	-0.119	-0.612	0.546
The rate of two-degree goiter	0.145	0.477	0.063	0.303	0.764
The rate of cretinism	0.114	0.193	0.125	0.590	0.561
UIC (μg/L) in school-aged children 8 to 10 years of age	-0.955	3.301	-0.054	-0.289	0.774
2012					
The rate of goiter	-2.606	4.642	-0.109	-0.561	0.579
The rate of two-degree goiter	-0.030	0.532	-0.012	-0.056	0.956
The rate of cretinism	0.174	0.174	0.209	1.000	0.328
2013					
The rate of goiter	-4.372	4.106	-0.204	-1.065	0.297
The rate of two-degree goiter	-0.058	0.529	-0.023	-0.109	0.914
The rate of cretinism	0.059	0.190	0.067	0.313	0.757

### Correlation between the content of iodine in soil and three endemic diseases

Table 3 shows the associations of the iodine content in soil with the goiter rate, two-degree goiter and cretinism in 2011, 2012 and 2013. Linear regression revealed no significant correlation between these data (*P* > 0.05).

**Table 3:** Correlations between the content of iodine in soil and three endemic diseases

Dependent variable	B	Std. Error	Beta	*t* value	*P* value
2011					
The rate of goiter	1.730	4.187	0.082	0.413	0.683
The rate of two-degree goiter	-0.439	0.467	-0.196	-0.940	0.358
The rate of cretinism	-0.194	0.159	-0.251	-1.215	0.237
2012					
The rate of goiter	1.846	4.150	0.089	0.445	0.660
The rate of two-degree goiter	-0.432	0.477	-0.190	-0.906	0.375
The rate of cretinism	-0.175	0.154	-0.235	-1.132	0.270
2013					
The rate of goiter	2.670	3.752	0.141	0.711	0.483
The rate of two-degree goiter	-0.259	0.454	-0.118	-0.572	0.573
The rate of cretinism	-0.164	0.151	-0.226	-1.088	0.288

#### Changes in the daily iodized intake and coverage rate of iodized salt

**Fig. 1:**
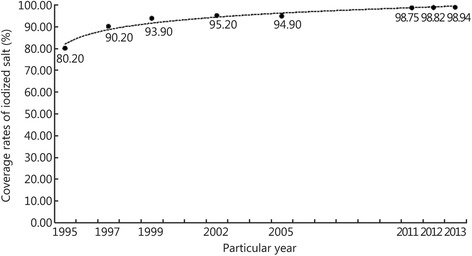
Changes in the coverage rate of iodized salt. The line graph describes the results of 5 cross-sectional and 3 consecutive studies in chronological order

Table 4 shows that one-way ANOVA revealed no differences in the daily iodized salt intake across the provinces in the three years (*F* = 0.886, *P* = 0.647). Figure 1 shows that the coverage rate of iodized salt exhibited large fluctuations in China. Since the implementation of USI in 1995, the coverage rate of iodized salt was increasing in successive years and exceeded 90% after 1997. From 2011 to 2013, the coverage rate of iodized salt stayed over 98.75%.

**Table 4:** Changes in the daily iodized salt intake

Year	Mean	Std. Deviation	Std. Error	*P* value	*F* value
2011 (*n* = 31)	12.8236	2.20918	0.39678	0.002	—
2012 (*n* = 31)	12.8477	2.19188	0.39367	0.003	—
2013 (*n* = 30^a^)	12.0787	2.02006	0.36881	0.176	—
Test of homogeneity of variances	—	—	—	0.692	0.370
ANOVA	—	—	—	0.647	0.886

^a^: There are 30 provinces because Shanghai provided no monitoring data in 2013

#### Goiter rates change in the three years

Table 5 shows that goiter rates were stable across the three years, and a decreasing trend was observed. Tibet exhibited a significant reduction in 2013 compared to the previous two years (2011 = 189.58‱, 2012 = 182.70‱, and 2013 = 132.79‱).

**Table 5:** Changes in goiter rates over three years

Region	The rate of goiter (‱^a^) in 2011	The rate of goiter (‱^a^) in 2012	The rate of goiter (‱^a^) in 2013
Total	36.92	36.78	35.48
Beijing	0.14	0.14	0.14
Tianjin	6.55	5.17	5.15
Hebei	12.26	11.50	11.50
Shanxi	11.64	11.40	10.56
Nei Monggol	61.33	55.42	55.40
Liaoning	30.26	30.35	29.72
Jilin	156.19	161.77	161.03
Heilongjiang	68.75	65.51	59.58
Shanghai	—	—	—
Jiangsu	46.07	42.99	33.84
Zhejiang	1.11	1.25	1.41
Anhui	14.97	14.97	4.06
Fujian	24.17	22.85	22.64
Jiangxi	89.72	90.65	89.74
Shandong	20.39	20.93	20.93
Henan	7.21	6.83	6.63
Hubei	24.97	29.64	29.21
Hunan	111.68	111.30	112.25
Guangdong	—	—	—
Guangxi	79.96	80.03	79.40
Hainan	7.22	2.37	0.43
Chongqing	41.74	39.89	42.43
Sichuan	12.01	11.91	11.91
Guizhou	83.95	81.02	80.81
Yunnan	3.84	3.56	3.44
Tibet	189.58	182.70	132.79
Shaanxi	105.44	104.25	103.32
Gansu	39.14	36.73	36.50
Qinghai	—	—	—
Ningxia	0.37	0.38	0.37
Xinjiang	23.38	22.48	22.23

‱^a^: Represent 1/10000 (‱) and data were not collected in Shanghai, Guangdong and Qinghai

## Discussion

The present study used national epidemiological survey data because the monitoring system of IDD in China is relatively precise for monitoring the iodized salt intake by county and monitoring the prevalence of IDD by province, which provided strong support for the 2011-2013 national monitoring data. Our work is different from studies with many on-site surveys; instead, it stands on the overall continuous levels to explore the influence of USI on the IDD and assess the status of table salt as the carrier for iodine fortification, which has crucial significance. This study is also a consecutive analysis in time, which is rarely analyzed in the substantial cross-sectional study literature. Goiter prevalence, UIC and salt iodine are 3 indicators that exhibit their own characteristics within certain time limits of each other. Consequently, the results of a cross-sectional survey reflect only the status of iodine nutrition and prevalence in individuals at that time. It has been well documented in previous studies that the prevalence of goiter is an index that changes relatively slowly and steadily [32, 33]. Several years or longer are needed to achieve a non-epidemic state in an endemic region of goiter caused by iodine deficiency after the implementation of full supplement conditions [32]. In contrast, iodized salt with UIC produces rapid changes that allow the iodine levels of the population to be instantly measured. Therefore, studies of the fluctuation and changes in the IDD prevalence and the correlations between IDD with iodized salt and UIC may induce errors based on the reliance of cross-sectional results. A longitudinal continuous observation of the results of 3 consecutive monitoring periods may identify changing trends and laws as well as the occurrence of the problem.

Our findings of the continued reduction of IDD in China are consistent with previous studies [14, 34]. There was no significant association between iodized salt intake and the three endemic diseases, which revealed that the current nutrition level of iodized salt did not cause the high goiter prevalence. Even when considering the confounders of goiter, such as genetic, metabolism, drugs, etc., the most important factor remains the lack of iodine. Therefore, in accordance with these results, the controversy that iodized salt increased the risk of thyroid disorders in China in the past decade is beginning to be cleared. We demonstrated no differences in the daily iodized salt intake across the provinces in three years using one-way ANOVA, which suggests that the daily iodized salt intake levels of residents in the three consecutive years remained stable. The results of our data demonstrated that the daily iodine salt intake values of a reference male were 12.63 g, 12.64 g and 12.23 g in 2011, 2012 and 2013, respectively. The current standard for salt iodization in China is 20-30 mg/kg according to the fourth adjustment of the iodine content of edible salt in 2010 [26]. Taking 2012 as an example, the daily iodine intake of a reference male ranged from 252.8 to 379.2 μg. The recommended dietary allowance of iodine for an adult male is 150 μg/d according to the WHO/UNICEF/ICCIDD [31], and the tolerable daily intake (TDI) of iodine is 600 μg/d according to the WHO [35]. These criteria indicate that the current iodine intake in China exceeded the recommended nutrient intake of WHO, but it was below the TDI, which may explain the present results that iodized salt intake was not associated with the three endemic diseases in question. However, this finding indicates that we still need to reduce the intake of iodized salt or the iodine content of iodized salt to achieve a more conducive level of iodine intake for the health of residents in China.

The UIC is currently the most practical biochemical marker for iodine nutrition [36]. Our results indicated that the present iodized salt intake may not be associated with UIC in Chinese children. We analyzed the fundamental reasons for this result and concluded that the per capita daily intake of iodized salt may not exactly reflect the iodine intake in children, and the UIC varied considerably in different regions, studies, and research methods. Urinary iodine excretion, as determined by the iodine/creatinine ratio, is influenced by iodized salt intake and additional factors, such as the muscle mass and physical activity [37, 38]. The examination of only one year of UIC may not precisely evaluate the iodine nutrition of subjects. Therefore, accurate correlations of iodized salt with UIC in school-aged children deserve more thorough investigation with large sample studies. Our findings indicated that the UIC of school children aged 8–10 years was 226.5 μg/L in 2011. This iodine nutrition is adequate according to the 2007 criteria of the WHO/UNICEF/ICCIDD for assessing iodine nutrition [17]. These results demonstrate that iodized salt intake did not affect the UIC in children or exceed the appropriate level of iodine nutrition in children.

Different environments have differences in the iodine intake. Iodine in the soil is absorbed into the human food chain through plants, which could affect the intake of iodine and occurrence of goiter. However, we did not observe a significant association between the iodine soil content and the three endemic diseases. Iodine in the soil may exert a small effect on iodine intake in China, but iodized salt is the main source of iodine in the Chinese population [39].

We demonstrated that the coverage rate of iodized salt increased annually to over 98.7% in 2011-2013 (Fig. 1). This result suggests that the government’s prevention and cure of IDD is improving, and the coverage rates gradually stabilized in 2011. Iodized salt has become the primary source of dietary iodine.

Unlike the coverage rate of iodized salt, we observed that goiter prevalence decreased continuously, while it remained stable for three years (Table 5). This result illustrates that the current nutrition level of iodized salt did not cause the high goiter prevalence and iodine fortification-induced goiter is not extensive. Our previous studies focused more on the rise in thyroid disorders, such as goiter, hyperthyroidism, hypothyroidism, thyroiditis and other autoimmune disorders, which increased significantly after the implementation of USI in 1995 [40]. However, there are numerous predisposing factors for autoimmune thyroid disease, including genetic susceptibility and environmental factors, which commonly affect the incidence of this disease, and no studies have reported that iodized salt increased the high-iodine goiter prevalence worldwide in the nearly 90-year history of iodized salt. The government also adjusted the iodine concentration in iodized salt four times to maintain iodine nutrition at an appropriate level. Therefore, the Chinese government should adhere to the policies of the USI [41] because iodized salt reduces goiter prevalence [14].

There are some limitations to the present study. First, we lost the data from individual provinces, and only one year of UIC data is available, which may have biased the comprehensive analysis. Second, our survey data included only three years, which may be too short to observe changes in goiter prevalence. Additionally, there were no monitoring results for thyroid carcinoma and hyperthyroidism, which were deemed too closely related to the USI in many studies. It was more accurate to collect data from each city for linear regression.

## Conclusions

The iodized salt may not increase the risk of the three endemic diseases at present. No statistical associations between iodized salt and the prevalence rates of goiter, two-degree goiter and cretinism were observed in Chinese populations. There were no significant relationships between the iodine soil content and the three endemic diseases in question. Non-iodized salt should be forbidden to improve the coverage rate of iodized salt, and this policy should be adopted to ensure that the UIC is maintained at an optimal level (100-199 μg/L). Future studies should monitor the local consumption rates of iodized salt to provide a more complete national database of iodine intake status. Our findings suggest that the USI policy should be enforced in China.

## Abbreviations

ANOVA: Analysis of variance
IDD: Iodine deficiency disorders
IED: Iodine excess disorders
PPS: Probability proportional-to-size
TDI: Tolerable daily intake
UIC: Urinary iodine concentration
USI: Universal salt iodization
WHO: World Health Organization
